# Crystal structure, Hirshfeld surface analysis and inter­action energy, DFT and anti­bacterial activity studies of (*Z*)-4-hexyl-2-(4-methyl­benzyl­idene)-2*H*-benzo[*b*][1,4]thia­zin-3(4*H*)-one

**DOI:** 10.1107/S205698902000657X

**Published:** 2020-05-22

**Authors:** Ghizlane Sebbar, Brahim Hni, Tuncer Hökelek, Joel T. Mague, Nada Kheira Sebbar, Bouchra Belkadi, El Mokhtar Essassi

**Affiliations:** a Laboratory of Microbiology and Molecular Biology, Faculty of Sciences, University Mohammed V, Rabat, Morocco; b Laboratoire de Chimie Organique Heterocyclique URAC 21, Pôle de Competence Pharmacochimie, Faculté des Sciences, Université Mohammed V, Rabat, Morocco; c Department of Physics, Hacettepe University, 06800 Beytepe, Ankara, Turkey; d Department of Chemistry, Tulane University, New Orleans, LA 70118, USA; e Laboratoire de Chimie Appliquée et Environnement, Equipe de Chimie Bioorganique Appliquée, Faculté des Sciences, Université Ibn Zohr, Agadir, Morocco

**Keywords:** crystal structure, hydrogen bond, benzo­thia­zine, anti­bacterial activity, Hirshfeld surface

## Abstract

The title compound, C_22_H_25_NOS, consists of methyl­benzyl­idene and benzo­thia­zine units linked to a hexyl moiety, where the thia­zine ring adopts a screw-boat conformation. In the crystal, inversion dimers are formed by weak C—H_Mthn_⋯O_Bnzthz_ hydrogen bonds and are linked into chains extending along the *a*-axis direction by weak C—H_Bnz_⋯O_Bnzthz_ (Bnz = benzene, Bnzthz = benzo­thia­zine and Mthn = methine) hydrogen bonds. A Hirshfeld surface analysis of the crystal structure indicates that the most important contributions for the crystal packing are from H⋯H (59.2%) and H⋯C/C⋯H (27.9%) inter­actions. Hydrogen bonding and van der Waals inter­actions are the dominant inter­actions in the crystal packing. Computational chemistry indicates that in the crystal, the C—H_Bnz_⋯O_Bnzthz_ and C—H_Mthn_⋯O_Bnzthz_ hydrogen-bond energies are 75.3 and 56.5 kJ mol^−1^, respectively. Density functional theory (DFT) optimized structures at the B3LYP/ 6–311 G(d,p) level are compared with the experimentally determined mol­ecular structure in the solid state. The HOMO—LUMO behaviour was elucidated to determine the energy gap. Moreover, the anti­bacterial activity of the title compound was evaluated against gram-positive and gram-negative bacteria.

## Abstract 2

The benzo­thia­zine moiety is folded along the N⋯S axis and a puckering analysis of the conformation of the heterocyclic ring was performed. The hexyl chain is mainly in an extended conformation. In the crystal, inversion dimers are formed by weak C—H_Mthn_⋯O_Bnzthz_ hydrogen bonds and are linked into chains extending along the *a*-axis direction by weak C—H_Bnz_⋯O_Bnzthz_ (Bnz = benzene, Bnzthz = benzo­thia­zine and Mthn = methine) hydrogen bonds.

## Chemical context   

1,4-Benzo­thia­zine derivatives constitute an important class of heterocyclic compounds which, even when part of a complex mol­ecule, possess a wide spectrum of biological activities (Sebbar *et al.*, 2016*a* ▸; Gupta *et al.*, 2009 ▸). Various 1,4-benzo­thia­zine derivatives have been synthesized by several methods (Parai & Panda, 2009 ▸; Barange *et al.*, 2007 ▸; Saadouni *et al.*, 2014 ▸). 1,4-Benzo­thia­zine derivatives are important because of their inter­esting biological properties such as anti-bacterial (Olayinka, 2012 ▸; Bhikan *et al.*, 2012 ▸), anti-fungal (Schiaffella *et al.*, 2006 ▸; Gupta & Wagh, 2006 ▸), anti­proliferative (Zieba *et al.*, 2010 ▸), anti­malarial (Baraza­rte *et al.*, 2009 ▸) and anti-inflammatory (Kaneko *et al.*, 2002 ▸) activities. The biological activities of some 1,4-benzo­thia­zines are similar to those of pheno­thia­zines, featuring the same structural specificity (Hni *et al.*, 2019**a*, ▸b*; Ellouz *et al.*, 2017*a* ▸,*b* ▸; Sebbar *et al.*, 2019*a* ▸,*b* ▸).

In a continuation of our research devoted to the development of substituted 1,4-benzo­thia­zine derivatives (Ellouz *et al.*, 2015 ▸, 2019 ▸; Sebbar *et al.*, 2015, 2017*a* ▸; Ellouz *et al.*), we have synthesized the title compound, **I**, by reaction of hexyl chloride with 2-(4- methyl­benzyl­idene)-3,4-di­hydro-2*H*-1,4-benzo­thia­zin-3 -one and potassium carbonate in the presence of tetra-*n*-butyl­ammonium bromide (as catalyst). We report herein the synthesis, the mol­ecular and crystal structures along with the Hirshfeld surface analysis and inter­action energy calculation [using CE–B3LYP/6–31G(d,p) energy model] and the density functional theory (DFT) computational calculation carried out at the B3LYP/6–311 G(d,p) level for comparing with the experimentally determined mol­ecular structure in the solid state of the title compound. Moreover, the anti­bacterial activity of **I** is evaluated against gram-positive and gram-negative bacteria (*viz.*, *Escherichia coli*, *Pseudomonas aeruginosa*, *Staphylococcus aureus* and *Streptococcus fasciens*).
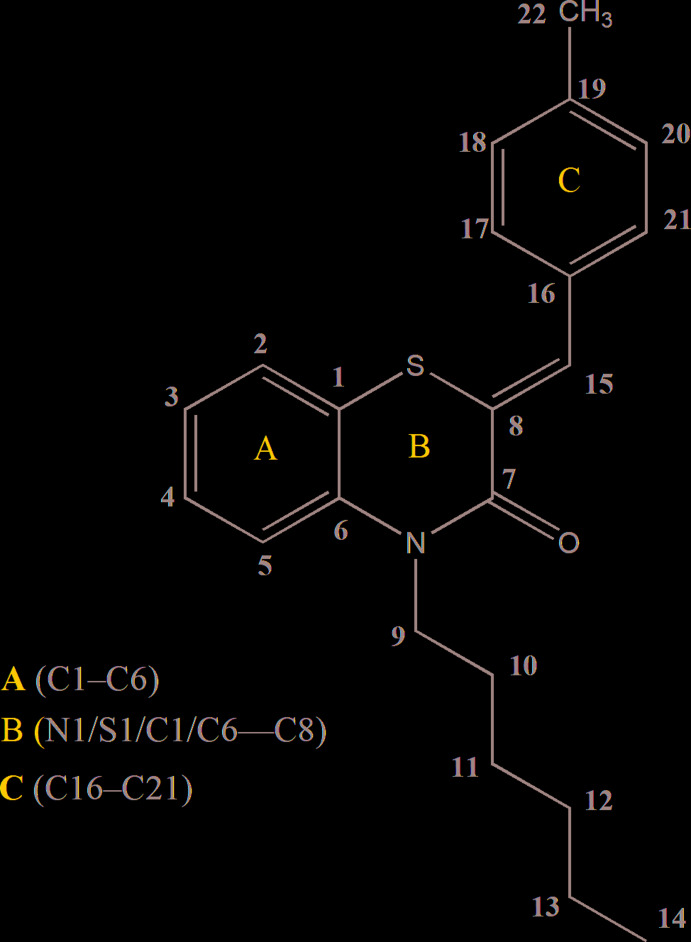


## Structural commentary   

**Figure 1:**
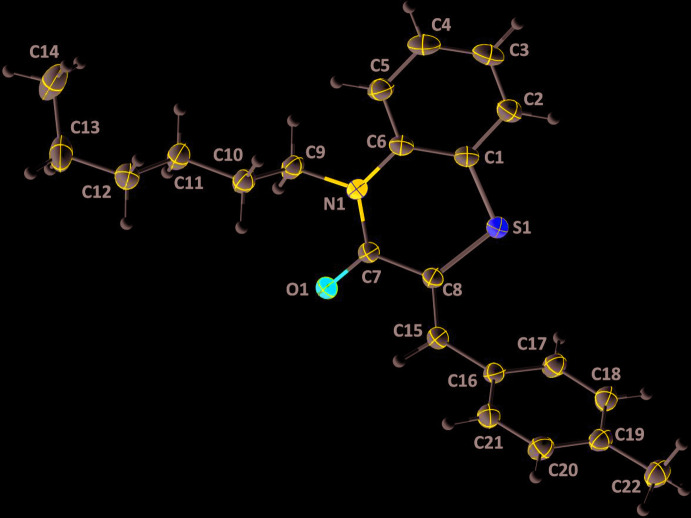
The asymmetric unit of the title compound with the atom-numbering scheme. Displacement ellipsoids are drawn at the 50% probability level.

The title compound, **I**, consists of methyl­benzyl­idene and benzo­thia­zine units linked to a hexyl moiety, where the thia­zine ring adopts a screw-boat conformation (Fig. 1 ▸). The heterocyclic portion of the benzo­thia­zine moiety is folded about the S1⋯N1 axis with the dihedral angle between the planes defined by N1/C7/C8/S1 and S1/C1/C6/N1 being 30.28 (6)°. A puckering analysis of the thia­zine, *B* (N1/S1/C1/C6–C8), ring conformation gave the parameters *Q*_T_ = 0.4853 (12) Å, θ = 69.48 (15)° and φ = 329.03 (18)°, indicating a screw-boat conformation. The dihedral angle between the benzene rings *A* (C1–C6) and *C* (C16–C21) is 75.64 (5)°. The base of the *n*-hexyl chain is approximately perpendicular to the mean plane of the benzo­thia­zine unit, as indicated by the C6—N1—C9—C10 torsion angle of −96.2 (1)°. The remainder of this chain, with the exception of the terminal methyl group, is in an extended conformation (Fig. 1 ▸).

## Supra­molecular features   

**Figure 2:**
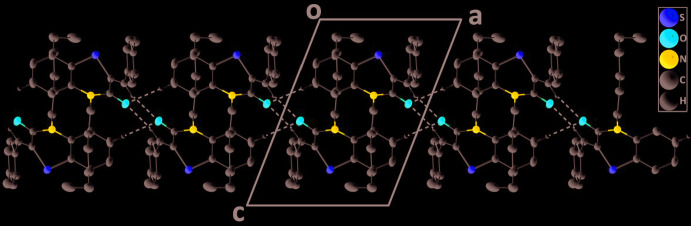
Detail of the chain of dimers viewed down the *b*-axis direction with the weak C—H_Mthn_⋯O_Bnzthz_ and C—H_Bnz_⋯O_Bnzthz_ (Bnz = benzene, Bnzthz = benzo­thia­zine and Mthn = methine) hydrogen bonds depicted by dashed lines.

In the crystal, inversion dimers are formed by weak C—H_Mthn_⋯O_Bnzthz_ hydrogen bonds (Table 1 ▸) and are linked into chains extending along the *a*-axis direction by weak C—H_Bnz_⋯O_Bnzthz_ hydrogen bonds (Table 1 ▸, Figs. 2 ▸ and 3 ▸) (Bnz = benzene, Bnzthz = benzo­thia­zine and Mthn = methine).

## Hirshfeld surface analysis   

**Figure 4:**
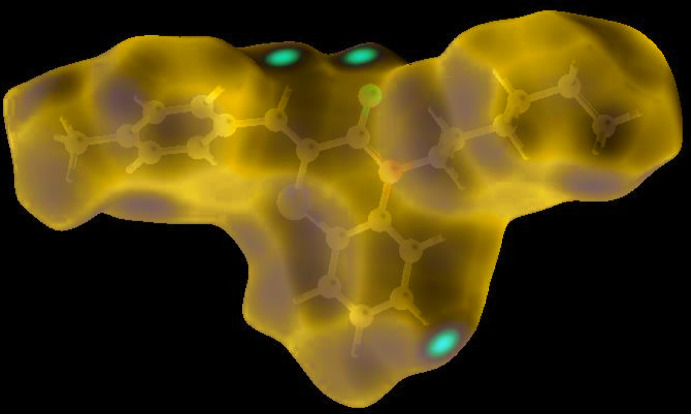
View of the three-dimensional Hirshfeld surface of the title compound plotted over *d*_norm_ in the range −0.2415 to 1.4195 a.u.

**Figure 5:**
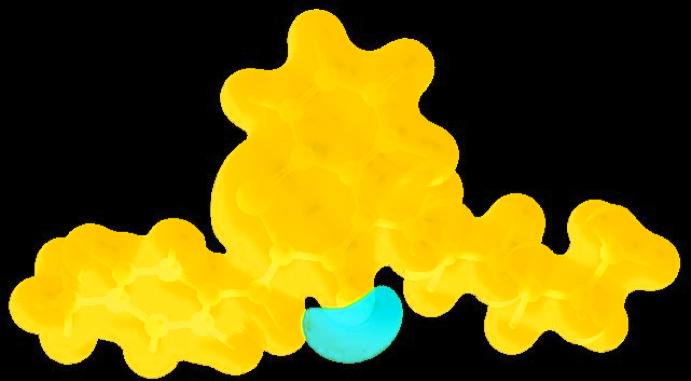
View of the three-dimensional Hirshfeld surface of the title compound plotted over electrostatic potential energy in the range −0.0500 to 0.0500 a.u. using the STO-3 G basis set at the Hartree–Fock level of theory hydrogen-bond donors and acceptors are shown as blue and red regions around the atoms corresponding to positive and negative potentials, respectively.

**Figure 6:**
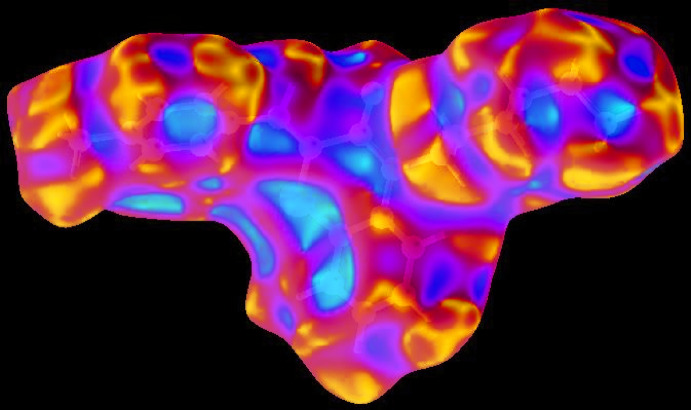
Hirshfeld surface of the title compound plotted over shape-index.

In order to visualize the inter­molecular inter­actions in the crystal of the title compound, a Hirshfeld surface (HS) analysis (Hirshfeld, 1977 ▸; Spackman & Jayatilaka, 2009 ▸) was carried out by using *Crystal Explorer 17.5* (Turner *et al.*, 2017 ▸). In the HS plotted over *d*_norm_ (Fig. 4 ▸), the white surface indicates contacts with distances equal to the sum of van der Waals radii, and the red and blue colours indicate distances shorter (in close contact) or longer (distinct contact) than the van der Waals radii, respectively (Venkatesan *et al.*, 2016 ▸). The bright-red spots appearing near O1 and hydrogen atoms H4 and H15 indicate their roles as the respective donors and/or acceptors; they also appear as blue and red regions corresponding to positive and negative potentials on the HS mapped over electrostatic potential (Spackman *et al.*, 2008 ▸; Jayatilaka *et al.*, 2005 ▸) as shown in Fig. 5 ▸. The blue regions indicate the positive electrostatic potential (hydrogen-bond donors), while the red regions indicate the negative electrostatic potential (hydrogen-bond acceptors). The shape-index of the HS is a tool to visualize the π–π stacking by the presence of adjacent red and blue triangles; if there are no adjacent red and/or blue triangles, then there are no π–π inter­actions. Fig. 6 ▸ clearly suggests that there are no π–π inter­actions in (I).

**Figure 7:**
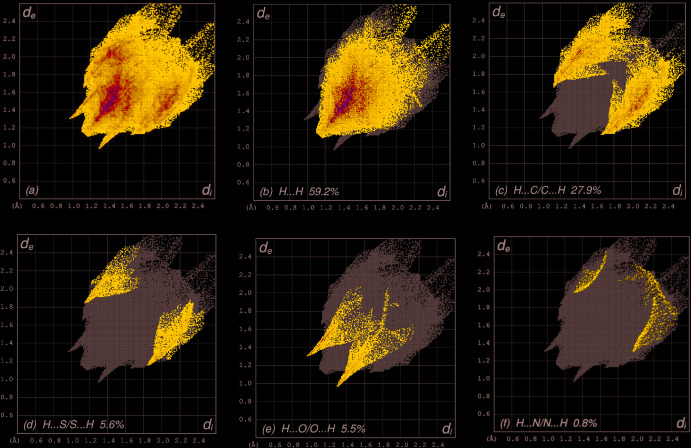
The full two-dimensional fingerprint plots for the title compound, showing (*a*) all inter­actions, and delineated into (*b*) H⋯H, (*c*) H⋯C/C⋯H, (*d*) H⋯S/S⋯H, (*e*) H⋯O/O⋯H and (*f*) H⋯N/N⋯H inter­actions. The *d*_i_ and *d*_e_ values are the closest inter­nal and external distances (in Å) from given points on the Hirshfeld surface contacts.

The overall two-dimensional fingerprint plot, Fig. 7 ▸ *a*, and those delineated into H⋯H, H⋯C/C⋯H, H⋯S/S⋯H, H⋯O/O⋯H and H⋯N/N⋯H contacts (McKinnon *et al.*, 2007 ▸) are illustrated in Fig. 7 ▸ *b-*-*f*, respectively, together with their relative contributions to the Hirshfeld surface. The most important inter­action is H⋯H, contributing 59.2% to the overall crystal packing, which is reflected in Fig. 7 ▸ *b* as widely scattered points of high density due to the large hydrogen content of the mol­ecule with the tip at *d*_e_ = *d*_i_ = 1.14 Å. In the absence of C—H⋯π inter­actions, the pair of characteristic wings in the fingerprint plot delineated into H⋯C/C⋯H contacts (Fig. 7 ▸ *c*, 27.9% contribution to the HS) has the tips at *d*_e_ + *d*_i_ = 2.77 Å. The pair of spikes in the fingerprint plot delineated into H⋯S/S⋯H (Fig. 7 ▸ *d*, 5.6% contribution) has the tips at *d*_e_ + *d*_i_ = 2.98 Å. The H⋯O/O⋯H contacts (Fig. 7 ▸ *e*, 5.5% contribution) have a symmetrical distribution of points with the tips at *d*_e_ + *d*_i_ = 2.27 Å. Finally, the H⋯N/N⋯H contacts (Fig. 7 ▸ *f)*, make only a 0.8% contribution to the HS with the tips at *d*_e_ + *d*_i_ = 3.28 Å.

**Figure 8:**
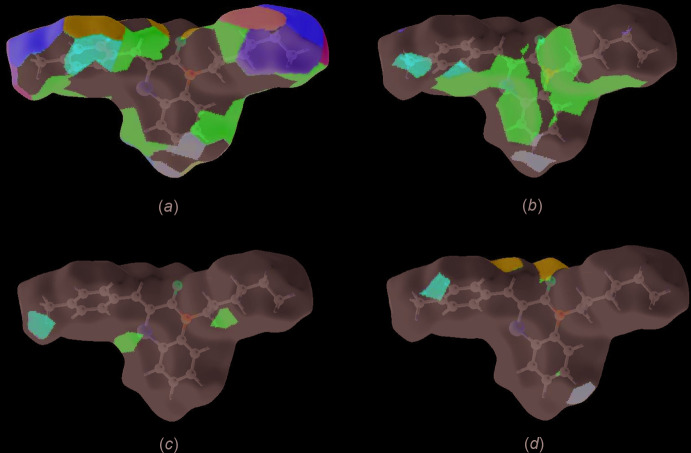
The Hirshfeld surface representations with the function *d*_norm_ plotted onto the surface for (*a*) H⋯H, (*b*) H⋯C/C⋯H, (*c*) H⋯S/S⋯H and (*d*) H⋯O/O⋯H inter­actions.

The Hirshfeld surface representations with the function *d*_norm_ plotted onto the surface are shown for the H⋯H, H⋯C/C⋯H, H⋯S/S⋯H and H⋯O/O⋯H inter­actions in Fig. 8 ▸ *a-*-*d*, respectively.

The Hirshfeld surface analysis confirms the importance of H-atom contacts in establishing the packing. The large number of H⋯H and H⋯C/C⋯H inter­actions suggest that van der Waals inter­actions and hydrogen bonding play the major roles in the crystal packing (Hathwar *et al.*, 2015 ▸).

## Inter­action energy calculations   

The inter­molecular inter­action energies are calculated using CE–B3LYP/6–31G(d,p) energy model available in *Crystal Explorer 17.5* (Turner *et al.*, 2017 ▸), where a cluster of mol­ecules is generated by applying crystallographic symmetry operations with respect to a selected central mol­ecule within the radius of 3.8 Å by default (Turner *et al.*, 2014 ▸). The total inter­molecular energy (*E*_tot_) is the sum of electrostatic (*E*_ele_), polarization (*E*_pol_), dispersion (*E*_dis_) and exchange-repulsion (*E*_rep_) energies (Turner *et al.*, 2015 ▸) with scale factors of 1.057, 0.740, 0.871 and 0.618, respectively (Mackenzie *et al.*, 2017 ▸). Hydrogen-bonding inter­action energies (in kJ mol^−1^) are −15.5 (*E*_ele_), −2.9 (*E*_pol_), −109.6 (*E*_dis_), 62.8 (*E*_rep_) and −75.3 (*E*_tot_) for C4—H4⋯O1 and −24.8 (*E*_ele_), −9.3 (*E*_pol_), −60.1 (*E*_dis_), 46.9 (*E*_rep_) and −56.5 (*E*_tot_) for C15—H15⋯O1.

## DFT calculations   

**Figure 9:**
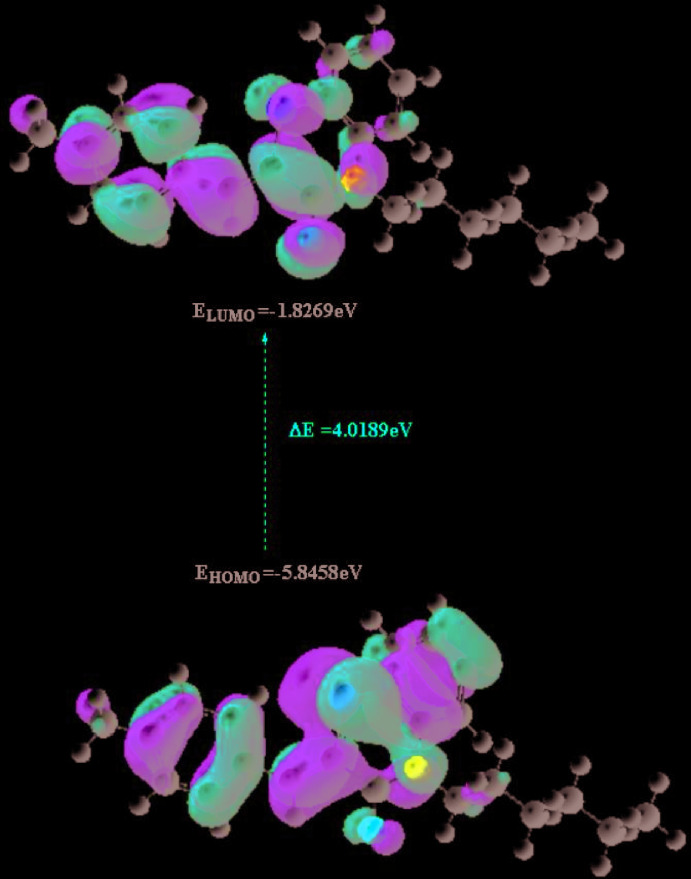
The energy band gap of the title compound.

The optimized structure of the title compound in the gas phase was generated theoretically *via* density functional theory (DFT) using standard B3LYP functional and 6–311 G(d,p) basis-set calculations (Becke, 1993 ▸) as implemented in *GAUSSIAN 09* (Frisch *et al.*, 2009 ▸). The theoretical and experimental results are in good agreement (Table 2 ▸). The highest-occupied mol­ecular orbital (HOMO), acting as an electron donor, and the lowest-unoccupied mol­ecular orbital (LUMO), acting as an electron acceptor, are very important parameters for quantum chemistry. When the energy gap is small, the mol­ecule is highly polarizable and has high chemical reactivity. The DFT calculations provide some important information on the reactivity and site selectivity of the mol­ecular framework. *E*_HOMO_ and *E*_LUMO_ clarify the inevitable charge-exchange collaboration inside the studied material, electronegativity (χ), hardness (η), potential (μ), electrophilicity (ω) and softness (*σ*) are recorded in Table 3 ▸. The significance of η and *σ* is for the evaluation of both the reactivity and stability. The electron transition from the HOMO to the LUMO energy level is shown in Fig. 9 ▸. The HOMO and LUMO are localized in the plane extending from the whole (Z)-2-(4-methyl­benzyl­idene)-4-hexyl-2*H*-benzo[*b*][1,4]thia­zin-3(4*H*)-one ring. The energy band gap [Δ*E* = *E*_LUMO_ − *E*_HOMO_] of the mol­ecule is 4.0189 eV, and the frontier mol­ecular orbital energies, *E*_HOMO_ and *E*_LUMO_ are −5.8458 and −1.8269 eV, respectively.

## Database survey   

A search in the Cambridge Structural Database (Groom *et al.*, 2016 ▸), for compounds containing the fragment **II** [Chem scheme2] (*R*_1_ = Ph, *R*_2_ = C), gave 15 hits, including with *R*_1_ = 4-ClC_6_H_4_ and *R*_2_ = CH_2_CH_2_CH_2_CH_3_ (**IIa** [Chem scheme2]) (Ellouz *et al.*, 2017*b* ▸), *R*_1_ = 2,4-Cl_2_C_6_H_3_ and *R*_2_ = CH_2_Ph2 (**IIb** [Chem scheme2]) (Sebbar *et al.*, 2019*b* ▸), and *R*_1_ = 2-ClC_6_H_4_, *R*_2_ = CH_2_C≡CH (**IIc** [Chem scheme2]) (Sebbar *et al.*, 2017*b* ▸), *R*_1_ = 4-FC_6_H_4_ and *R*_2_ = CH_2_C≡CH (**IIc** [Chem scheme2]) (Hni *et al.*,2019*a* ▸), CH_2_COOH (Sebbar *et al.*, 2016*a* ▸), *R*_1_ = 2,4-Cl_2_C_6_H_3_ and *R*_2_ = (CH_2_)_8_CH_3_ (Hni *et al.*, 2020 ▸), *R*_1_ = 4-ClC_6_H_4_ and *R*_2_ = CH_2_Ph2 (**IIb** [Chem scheme2]) (Ellouz *et al.*, 2016 ▸), *R*_1_ = 4-ClC_6_H_4_ and *R*_2_ = (**IId** [Chem scheme2]) (Ellouz *et al.*, 2017*a* ▸) or CH_2_C≡CH (**IIc**) (Sebbar *et al.*, 2014 ▸), *R*_1_ = 2,4-Cl_2_C_6_H_3_ and *R*_2_ = **IId** [Chem scheme2] (Hni *et al.*, 2019*b* ▸), *R*_1_ = 2,4-Cl_2_C_6_H_3_ and *R*_2_ =CH_2_CH_2_CN (**IIe** [Chem scheme2]) (Sebbar *et al.*, 2019*a* ▸), **IIf** [Chem scheme2] (Sebbar *et al.*, 2016*b* ▸) and **IIg** [Chem scheme2] (Ellouz *et al.*, 2015 ▸).

In the majority of these, the thia­zine ring is significantly folded about the S⋯N axis with dihedral angles between the two S/C/C/N planes ranging from *ca* 35° [**IIf** [Chem scheme2] (Sebbar *et al.*, 2016*b* ▸) and **IId** [Chem scheme2] (Ellouz *et al.*, 2017*a* ▸)] to *ca* 27° [**IIc** [Chem scheme2] (Hni *et al.*, 2019*a* [Chem scheme2] ▸) and **IIc** (Sebbar *et al.*, 2014 ▸)].

## Anti­bacterial activity   

**Figure 10:**
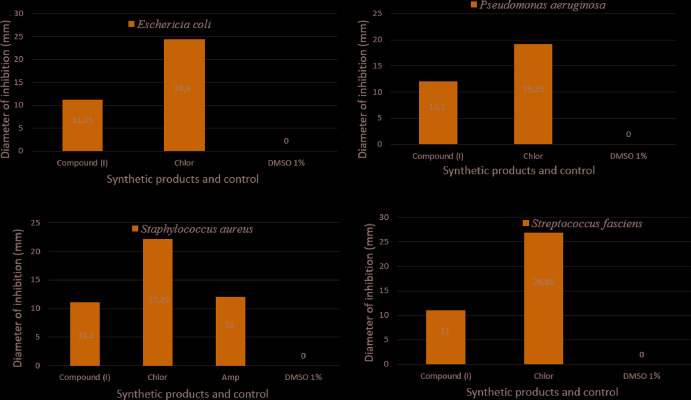
Anti­bacterial activity of the title compound (**I**) and commercial anti­biotic Chloramphenicol (Chlor) against bacteria *Escherichia coli*, *Pseudomonas aeruginosa*, *Staphylococcus aureus* and *Streptococcus fasciens*.

To compare and analyse the anti­bacterial behaviour of the title compound and commercial anti­biotics such as Chloramphenicol (Chlor), we have tested **I** against *Escherichia coli* 
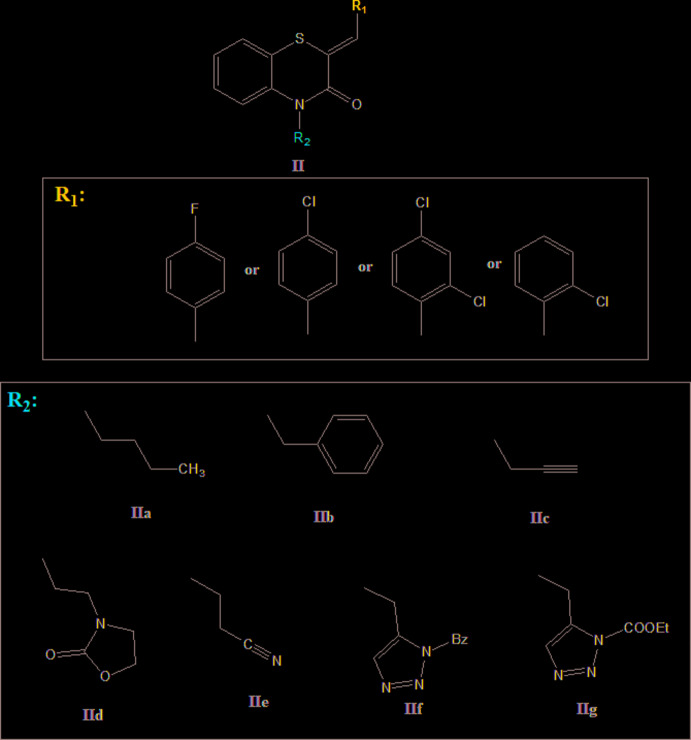


(ATTC-25922), *Pseudomonas aeruginosa* (ATCC-27853), *Staphylococcus aureus* (ATCC-25923) and *Streptococcus fasciens* (ATCC-29212) strains of bacteria using the diffusion method disk for evaluating the applicability of **I** as an anti­bacterial agent (Mabkhot *et al.*, 2016 ▸; Hoffmann *et al.*, 2017 ▸). Fig. 10 ▸ summarizes the diameter of inhibition (mm) values of **I** and the commercial anti­biotic Chlor. The determination of the minimum inhibition concentration MIC values of **I** against the bacteria are presented in Table 4 ▸. The results of the anti­bacterial activity of the product **I** obtained by the alkyl­ation reaction under the conditions of catalysis by liquid–solid phase transfer of hexyl chloride with 2-(4-methyl­benzyl­idene)-3,4-di­hydro-2*H*-1,4-benzo­thia­zin-3-one showed increases of MIC = 20 µg ml^−1^ for *Staphylococcus aureus*, MIC = 10 µg ml^−1^ for *Escherichia coli* and *Pseudomonas aeruginosa* and MIC = 5 µg ml^−1^ for *Streptococcus fasciens*, which corresponds to the best MIC activity as compared to the commercial anti­biotic. In addition, the maximum effect of **I** was recorded against *Pseudomonas aeruginosa* (diameter of inhibition 12.1 mm). Chlor presents an anti­bacterial activity diameter of inhibition of between 19 mm and 27 mm and no zone inhibition was observed with di­methyl­sulfoxide (DMSO) [(1%): 1 mL of DMSO added to 99 mL ofulltra-pure water] [The test samples were first dissolved in DMSO (1%), which did not affect the microbial growth.] On one hand, the chemical structure of **I** can explain this biological effect. The mechanism of action of **I** is not attributable to one specific mechanism, but there are several targets in the cell: degradation of the cell wall, damage to membrane proteins, damage to cytoplasmic membrane, leakage of cell contents and coagulation of cytoplasm. On the other hand, it should be noted that the functionalized derivatives by ester groups and benzene rings have the highest anti­bacterial coefficient (92% of pathogenic bacteria are sensitive). This study is expected to take anti-inflammatory, anti­fungal, anti-parasitic and anti-cancer activities, because the literature gives a lot of inter­esting results on these topics. Some other types of bacteria may possibly be tested by employing the same method so as eventually to generalize the suggested investigation method (Alderman & Smith, 2001 ▸).

## Synthesis and crystallization   

To a solution of 2-(4-methyl­benzyl­idene)-3,4-di­hydro-2*H*-1,4-benzo­thia­zin-3-one (0.70 g, 2 mmol), potassium carbonate (4 mmol) and tetra-*n*-butyl ammonium bromide (0.2 mmol) in DMF (15 ml) was added 1-chloro­hexane (0.48 g, 4 mmol). Stirring was continued at room temperature for 12 h. The reaction mixture was filtered and the solvent was removed. The residue was extracted with water. The organic compound was chromatographed on a column of silica gel using the mixture ethyl acetate–hexane (9:1) as eluent. Colourless crystals of the title compound **I** [Chem scheme1], were isolated when the solvent was allowed to evaporate (yield: 60%), m.p. > 284 K.

^1^H NMR (300 MHz, DMSO-*d*_6_) δ ppm: 0.88 (*t*, 3H, –CH_2_–CH_3_, *J* = 6.3 Hz); 2.37 (*s*, 3H, =CH-C_6_H_4_–CH_3_); 2.37–2.52 (*m*, 8H, 4CH_2_); 4.08 (*t*, 2H, N-CH_2_, *J* = 7.1 Hz); 7.06–7.57 (*m*, 8H, CH_arom_); 7.77 (*s*, 1H; =CH–C_6_H_4_Cl); ^13^C NMR (62.5 MHz, DMSO-*d*_6_) δ ppm: 13.86 (–CH_2_–CH3); 20.98 (=CH–C_6_H_4_–CH_3_); 22.02, 25,83 26.48, 30.84, (CH_2_); 44.24 (NCH_2_); 117.26, 123.47, 126.36, 127.55, 129.15, 129.15, 130.03, 130.03, (CH_arom_); 133.77 (CH_all­yl_); 118.5, 119.31, 131.39, 135.77, 139.92, (Cq); 160.48 (C=O).

## Refinement   

The experimental details including the crystal data, data collection and refinement are summarized in Table 5 ▸. The C-bound H atoms were positioned geometrically, with C—H = 0.95 Å (for aromatic and methine H atoms), 0.99 Å (for methyl­ene H atoms) and 0.98 Å (for methyl H atoms), and constrained to ride on their parent atoms, with *U*_iso_(H) = *k* × *U*_eq_(C), where *k* = 1.5 (for methyl H atoms) and *k* = 1.2 for other H atoms.

## Supplementary Material

Crystal structure: contains datablock(s) I, global. DOI: 10.1107/S205698902000657X/ex2032sup1.cif

Structure factors: contains datablock(s) I. DOI: 10.1107/S205698902000657X/ex2032Isup2.hkl

Supporting information file. DOI: 10.1107/S205698902000657X/ex2032Isup3.cdx

Supporting information file. DOI: 10.1107/S205698902000657X/ex2032Isup4.cml

CCDC reference: 2004559

Additional supporting information:  crystallographic information; 3D view; checkCIF report

## Figures and Tables

**Figure 3:**
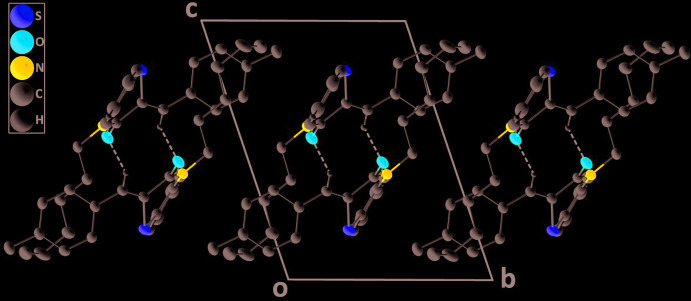
A partial packing diagram down the *a*-axis direction giving an end view of three adjacent chains.

**Table 1:** Hydrogen-bond geometry (Å, °)

*D*—H⋯*A*	*D*—H	H⋯*A*	*D*⋯*A*	*D*—H⋯*A*
C4—H4⋯O1^i^	0.95	2.42	3.349 (2)	168
C15—H15⋯O1^ii^	0.95	2.45	3.2977 (17)	148

Symmetry codes: (i) 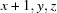; (ii) 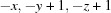.

**Table 2:** Comparison of the selected (X-ray and DFT) geometric data (Å, °)

Bonds/angles	X-ray	B3LYP/6–311G(d,p)
S1—C8	1.7552 (13)	1.83796
S1—C1	1.7560 (14)	1.83324
O1—C7	1.2310 (15)	1.25561
N1—C7	1.3687 (17)	1.39823
N1—C6	1.4207 (17)	1.42632
N1—C9	1.4756 (17)	1.48630
C1—C2	1.3928 (19)	1.39451
C1—C6	1.3976 (19)	1.40423
C2—C3	1.380 (2)	1.39431
C3—C4	1.379 (2)	1.39578
C4—C5	1.387 (2)	1.39431
		
C8—S1—C1	99.20 (6)	99.87
C7—N1—C6	124.55 (11)	124.76
C7—N1—C9	115.97 (11)	116.02
C6—N1—C9	119.35 (11)	119.89
C2—C1—C6	120.33 (13)	120.89
C2—C1—S1	117.86 (11)	118.13
C6—C1—S1	121.81 (10)	121.30
C3—C2—C1	120.67 (14)	120.36
C4—C3—C2	119.39 (14)	120.16

**Table 3:** Calculated energies

Mol­ecular Energy (a.u.) (eV)	Compound **I**
Total Energy, *TE* (eV)	−37591.8507
*E*_HOMO_ (eV)	−5.8458
*E*_LUMO_ (eV)	−1.8269
Gap, *ΔE* (eV)	4.0189
Dipole moment, *μ* (Debye)	2.5702
Ionization potential, *I* (eV)	5.8458
Electron affinity, *A*	1.8269
Electronegativity, *χ*	3.8363
Hardness, *η*	2.0095
Electrophilicity index, *ω*	3.6621
Softness, *σ*	0.4976
Fraction of electron transferred, *ΔN*	0.7872

**Table 4:** Minimal inhibitory concentration [MIC (μg/ml)] of the title compound **I** ATTC-25922 = *Escherichia coli*, ATCC-27853 = *Pseudomonas aeruginosa*, ATCC-25923 = *Staphylococcus aureus*, ATCC-29212 = *Streptococcus fasciens* and Chlor = Chloramphenicol.

Product	**I**	Chlor	DMSO
ATCC-25922	10	6.25	0
ATTC-25953	10	6.25	0
ATCC-27823	20	12.5	0
ATCC-29212	5	12.5	0

**Table 5:** Experimental details

Crystal data	
Chemical formula	C_22_H_25_NOS
*M*_r_	351.49
Crystal system, space group	Triclinic, *P* 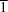
Temperature (K)	150
*a*, *b*, *c* (Å)	8.8581 (19), 9.183 (2), 13.021 (3)
α, β, γ (°)	106.474 (3), 109.398 (3), 93.383 (3)
*V* (Å^3^)	944.2 (4)
*Z*	2
Radiation type	Mo *K*α
μ (mm^−1^)	0.18
Crystal size (mm)	0.33 × 0.26 × 0.10

Data collection	
Diffractometer	Bruker Smart *APEX* CCD
Absorption correction	Multi-scan (*SADABS*; Krause *et al.*, 2015 ▸)
*T*_min_, *T*_max_	0.83, 0.98
No. of measured, independent and observed [*I* > 2σ(*I*)] reflections	17663, 4860, 3807
*R*_int_	0.029
(sin θ/λ)_max_ (Å^−1^)	0.677

Refinement	
*R*[*F* ^2^ > 2σ(*F* ^2^)], *wR*(*F* ^2^), *S*	0.046, 0.134, 1.08
No. of reflections	4860
No. of parameters	228
H-atom treatment	H-atom parameters constrained
Δρ_max_, Δρ_min_ (e Å^−3^)	0.54, −0.22

Computer programs: *APEX3* and *SAINT* (Bruker, 2016 ▸), *SHELXT2014/5* (Sheldrick, 2015*a* ▸), *SHELXL2018/3* (Sheldrick, 2015*b* ▸), *DIAMOND* (Brandenburg & Putz, 2012 ▸) and *SHELXTL* (Sheldrick, 2008 ▸).

## supplementary crystallographic information

### Crystal data

**Table:**

C_22_H_25_NOS	*Z* = 2
*M**_r_* = 351.49	*F*(000) = 376
Triclinic, *P*1	*D*_x_ = 1.236 Mg m^−^^3^
*a* = 8.8581 (19) Å	Mo *K*α radiation, λ = 0.71073 Å
*b* = 9.183 (2) Å	Cell parameters from 7052 reflections
*c* = 13.021 (3) Å	θ = 2.4–28.7°
α = 106.474 (3)°	µ = 0.18 mm^−^^1^
β = 109.398 (3)°	*T* = 150 K
γ = 93.383 (3)°	Plate, colourless
*V* = 944.2 (4) Å^3^	0.33 × 0.26 × 0.10 mm

### Data collection

**Table:**

Bruker Smart APEX CCD diffractometer	4860 independent reflections
Radiation source: fine-focus sealed tube	3807 reflections with *I* > 2σ(*I*)
Graphite monochromator	*R*_int_ = 0.029
Detector resolution: 8.3333 pixels mm^-1^	θ_max_ = 28.8°, θ_min_ = 1.8°
φ and ω scans	*h* = −11→11
Absorption correction: multi-scan (*SADABS*; Krause *et al.*, 2015)	*k* = −12→12
*T*_min_ = 0.83, *T*_max_ = 0.98	*l* = −17→17
17663 measured reflections	

### Refinement

**Table:**

Refinement on *F*^2^	Primary atom site location: dual
Least-squares matrix: full	Secondary atom site location: difference Fourier map
*R*[*F*^2^ > 2σ(*F*^2^)] = 0.046	Hydrogen site location: inferred from neighbouring sites
*wR*(*F*^2^) = 0.134	H-atom parameters constrained
*S* = 1.08	*w* = 1/[σ^2^(*F*_o_^2^) + (0.0868*P*)^2^] where *P* = (*F*_o_^2^ + 2*F*_c_^2^)/3
4860 reflections	(Δ/σ)_max_ = 0.002
228 parameters	Δρ_max_ = 0.54 e Å^−^^3^
0 restraints	Δρ_min_ = −0.22 e Å^−^^3^

### Special details

**Table:**

Experimental. The diffraction data were obtained from 3 sets of 400 frames, each of width 0.5° in ω, collected at φ = 0.00, 90.00 and 180.00° and 2 sets of 800 frames, each of width 0.45° in φ, collected at ω = –30.00 and 210.00°. The scan time was 20 sec/frame.
Geometry. All esds (except the esd in the dihedral angle between two l.s. planes) are estimated using the full covariance matrix. The cell esds are taken into account individually in the estimation of esds in distances, angles and torsion angles; correlations between esds in cell parameters are only used when they are defined by crystal symmetry. An approximate (isotropic) treatment of cell esds is used for estimating esds involving l.s. planes.
Refinement. Refinement of F^2^ against ALL reflections. The weighted R-factor wR and goodness of fit S are based on F^2^, conventional R-factors R are based on F, with F set to zero for negative F^2^. The threshold expression of F^2^ > 2sigma(F^2^) is used only for calculating R-factors(gt) etc. and is not relevant to the choice of reflections for refinement. R-factors based on F^2^ are statistically about twice as large as those based on F, and R- factors based on ALL data will be even larger. H-atoms attached to carbon were placed in calculated positions (C—H = 0.95 - 0.99 Å). All were included as riding contributions with isotropic displacement parameters 1.2 - 1.5 times those of the attached atoms.

### Fractional atomic coordinates and isotropic or equivalent isotropic displacement parameters (Å^2^)

**Table:**

	*x*	*y*	*z*	*U*_iso_*/*U*_eq_	
S1	0.48853 (4)	0.62268 (4)	0.80836 (3)	0.02689 (12)	
O1	0.14092 (11)	0.34562 (11)	0.54654 (8)	0.0296 (2)	
N1	0.40988 (13)	0.34294 (13)	0.59158 (9)	0.0230 (2)	
C1	0.62657 (16)	0.53904 (15)	0.74903 (11)	0.0238 (3)	
C2	0.79091 (16)	0.60067 (17)	0.80699 (13)	0.0295 (3)	
H2	0.824726	0.683588	0.877249	0.035*	
C3	0.90511 (17)	0.54252 (18)	0.76340 (14)	0.0344 (3)	
H3	1.017293	0.583176	0.804334	0.041*	
C4	0.85491 (18)	0.42485 (19)	0.65987 (14)	0.0359 (4)	
H4	0.932696	0.386664	0.628234	0.043*	
C5	0.69171 (17)	0.36181 (17)	0.60156 (13)	0.0304 (3)	
H5	0.658838	0.281107	0.530152	0.037*	
C6	0.57519 (15)	0.41547 (15)	0.64649 (11)	0.0230 (3)	
C7	0.28144 (15)	0.41309 (15)	0.60267 (11)	0.0221 (3)	
C8	0.31493 (15)	0.57412 (15)	0.68247 (11)	0.0229 (3)	
C9	0.36834 (18)	0.18446 (15)	0.51035 (12)	0.0272 (3)	
H9A	0.278067	0.127685	0.519876	0.033*	
H9AB	0.463261	0.131943	0.530044	0.033*	
C10	0.31894 (18)	0.17657 (16)	0.38509 (12)	0.0292 (3)	
H10A	0.222119	0.226310	0.364140	0.035*	
H10B	0.408117	0.234298	0.375094	0.035*	
C11	0.28073 (19)	0.01127 (16)	0.30521 (12)	0.0321 (3)	
H11A	0.381565	−0.033721	0.320101	0.038*	
H11B	0.202450	−0.049348	0.323086	0.038*	
C12	0.2103 (2)	−0.00319 (18)	0.17853 (13)	0.0370 (4)	
H12A	0.111018	0.044208	0.164526	0.044*	
H12B	0.289671	0.056538	0.160940	0.044*	
C13	0.1679 (2)	−0.1666 (2)	0.09621 (15)	0.0470 (4)	
H13A	0.112018	−0.164616	0.017068	0.056*	
H13B	0.090849	−0.227440	0.114929	0.056*	
C14	0.3135 (3)	−0.2469 (2)	0.09928 (16)	0.0522 (5)	
H14A	0.278359	−0.348108	0.039587	0.078*	
H14B	0.362499	−0.259934	0.174847	0.078*	
H14C	0.393526	−0.184479	0.085374	0.078*	
C15	0.20651 (16)	0.66746 (15)	0.65716 (11)	0.0240 (3)	
H15	0.112596	0.621988	0.589786	0.029*	
C16	0.21350 (15)	0.82914 (15)	0.71914 (11)	0.0237 (3)	
C17	0.28006 (17)	0.89520 (16)	0.83901 (11)	0.0270 (3)	
H17	0.320387	0.832386	0.885317	0.032*	
C18	0.28781 (18)	1.05119 (16)	0.89087 (12)	0.0299 (3)	
H18	0.332476	1.093311	0.972370	0.036*	
C19	0.23138 (17)	1.14756 (16)	0.82588 (12)	0.0275 (3)	
C20	0.16120 (18)	1.08124 (16)	0.70733 (13)	0.0301 (3)	
H20	0.119719	1.144064	0.661268	0.036*	
C21	0.15056 (17)	0.92502 (16)	0.65499 (12)	0.0287 (3)	
H21	0.099424	0.882241	0.573798	0.034*	
C22	0.2467 (2)	1.31839 (17)	0.88163 (14)	0.0363 (4)	
H22A	0.172104	1.360783	0.827572	0.054*	
H22B	0.358422	1.368479	0.902762	0.054*	
H22C	0.219515	1.337033	0.950821	0.054*	

### Atomic displacement parameters (Å^2^)

**Table:**

	*U*^11^	*U*^22^	*U*^33^	*U*^12^	*U*^13^	*U*^23^
S1	0.02168 (18)	0.0295 (2)	0.02434 (19)	0.00586 (14)	0.00650 (13)	0.00294 (14)
O1	0.0212 (5)	0.0261 (5)	0.0333 (5)	0.0031 (4)	0.0066 (4)	0.0017 (4)
N1	0.0227 (5)	0.0209 (6)	0.0238 (5)	0.0061 (4)	0.0084 (4)	0.0044 (4)
C1	0.0223 (6)	0.0248 (7)	0.0268 (7)	0.0070 (5)	0.0094 (5)	0.0109 (5)
C2	0.0237 (7)	0.0299 (7)	0.0330 (8)	0.0035 (6)	0.0068 (6)	0.0118 (6)
C3	0.0198 (6)	0.0420 (9)	0.0450 (9)	0.0073 (6)	0.0106 (6)	0.0203 (7)
C4	0.0270 (7)	0.0445 (9)	0.0459 (9)	0.0153 (7)	0.0198 (7)	0.0193 (8)
C5	0.0292 (7)	0.0334 (8)	0.0321 (7)	0.0123 (6)	0.0145 (6)	0.0103 (6)
C6	0.0213 (6)	0.0248 (7)	0.0254 (6)	0.0067 (5)	0.0084 (5)	0.0112 (5)
C7	0.0217 (6)	0.0214 (6)	0.0230 (6)	0.0049 (5)	0.0088 (5)	0.0060 (5)
C8	0.0200 (6)	0.0222 (6)	0.0249 (6)	0.0025 (5)	0.0088 (5)	0.0048 (5)
C9	0.0327 (7)	0.0200 (6)	0.0293 (7)	0.0083 (5)	0.0121 (6)	0.0070 (5)
C10	0.0353 (7)	0.0238 (7)	0.0276 (7)	0.0090 (6)	0.0109 (6)	0.0067 (6)
C11	0.0399 (8)	0.0255 (7)	0.0291 (7)	0.0072 (6)	0.0134 (6)	0.0050 (6)
C12	0.0375 (8)	0.0348 (8)	0.0310 (8)	0.0102 (7)	0.0074 (6)	0.0042 (6)
C13	0.0450 (10)	0.0445 (10)	0.0371 (9)	−0.0097 (8)	0.0171 (7)	−0.0073 (8)
C14	0.0842 (14)	0.0342 (9)	0.0395 (10)	0.0190 (9)	0.0272 (10)	0.0063 (7)
C15	0.0224 (6)	0.0232 (7)	0.0247 (6)	0.0037 (5)	0.0081 (5)	0.0060 (5)
C16	0.0206 (6)	0.0214 (6)	0.0283 (7)	0.0047 (5)	0.0097 (5)	0.0056 (5)
C17	0.0318 (7)	0.0247 (7)	0.0272 (7)	0.0084 (6)	0.0121 (6)	0.0099 (6)
C18	0.0337 (7)	0.0278 (7)	0.0256 (7)	0.0067 (6)	0.0106 (6)	0.0048 (6)
C19	0.0274 (7)	0.0219 (7)	0.0336 (7)	0.0047 (5)	0.0135 (6)	0.0067 (6)
C20	0.0328 (7)	0.0251 (7)	0.0342 (8)	0.0088 (6)	0.0115 (6)	0.0126 (6)
C21	0.0303 (7)	0.0263 (7)	0.0254 (7)	0.0061 (6)	0.0065 (5)	0.0063 (6)
C22	0.0433 (9)	0.0233 (7)	0.0397 (9)	0.0074 (6)	0.0150 (7)	0.0063 (6)

### Geometric parameters (Å, º)

**Table:**

S1—C8	1.7552 (13)	C11—H11B	0.9900
S1—C1	1.7560 (14)	C12—C13	1.515 (2)
O1—C7	1.2310 (15)	C12—H12A	0.9900
N1—C7	1.3687 (17)	C12—H12B	0.9900
N1—C6	1.4207 (17)	C13—C14	1.518 (3)
N1—C9	1.4756 (17)	C13—H13A	0.9900
C1—C2	1.3928 (19)	C13—H13B	0.9900
C1—C6	1.3976 (19)	C14—H14A	0.9800
C2—C3	1.380 (2)	C14—H14B	0.9800
C2—H2	0.9500	C14—H14C	0.9800
C3—C4	1.379 (2)	C15—C16	1.4615 (18)
C3—H3	0.9500	C15—H15	0.9500
C4—C5	1.387 (2)	C16—C21	1.3963 (18)
C4—H4	0.9500	C16—C17	1.4004 (18)
C5—C6	1.3958 (19)	C17—C18	1.3862 (19)
C5—H5	0.9500	C17—H17	0.9500
C7—C8	1.4922 (18)	C18—C19	1.395 (2)
C8—C15	1.3464 (18)	C18—H18	0.9500
C9—C10	1.5205 (19)	C19—C20	1.387 (2)
C9—H9A	0.9900	C19—C22	1.5067 (19)
C9—H9AB	0.9900	C20—C21	1.3851 (19)
C10—C11	1.5201 (19)	C20—H20	0.9500
C10—H10A	0.9900	C21—H21	0.9500
C10—H10B	0.9900	C22—H22A	0.9800
C11—C12	1.520 (2)	C22—H22B	0.9800
C11—H11A	0.9900	C22—H22C	0.9800
			
C8—S1—C1	99.20 (6)	C13—C12—C11	115.01 (14)
C7—N1—C6	124.55 (11)	C13—C12—H12A	108.5
C7—N1—C9	115.97 (11)	C11—C12—H12A	108.5
C6—N1—C9	119.35 (11)	C13—C12—H12B	108.5
C2—C1—C6	120.33 (13)	C11—C12—H12B	108.5
C2—C1—S1	117.86 (11)	H12A—C12—H12B	107.5
C6—C1—S1	121.81 (10)	C12—C13—C14	113.96 (15)
C3—C2—C1	120.67 (14)	C12—C13—H13A	108.8
C3—C2—H2	119.7	C14—C13—H13A	108.8
C1—C2—H2	119.7	C12—C13—H13B	108.8
C4—C3—C2	119.39 (14)	C14—C13—H13B	108.8
C4—C3—H3	120.3	H13A—C13—H13B	107.7
C2—C3—H3	120.3	C13—C14—H14A	109.5
C3—C4—C5	120.51 (13)	C13—C14—H14B	109.5
C3—C4—H4	119.7	H14A—C14—H14B	109.5
C5—C4—H4	119.7	C13—C14—H14C	109.5
C4—C5—C6	120.83 (14)	H14A—C14—H14C	109.5
C4—C5—H5	119.6	H14B—C14—H14C	109.5
C6—C5—H5	119.6	C8—C15—C16	128.87 (12)
C5—C6—C1	118.17 (12)	C8—C15—H15	115.6
C5—C6—N1	120.84 (12)	C16—C15—H15	115.6
C1—C6—N1	120.95 (12)	C21—C16—C17	117.48 (12)
O1—C7—N1	120.68 (12)	C21—C16—C15	118.10 (12)
O1—C7—C8	120.59 (11)	C17—C16—C15	124.42 (12)
N1—C7—C8	118.71 (11)	C18—C17—C16	120.77 (12)
C15—C8—C7	118.49 (12)	C18—C17—H17	119.6
C15—C8—S1	124.93 (10)	C16—C17—H17	119.6
C7—C8—S1	116.43 (9)	C17—C18—C19	121.36 (13)
N1—C9—C10	113.78 (11)	C17—C18—H18	119.3
N1—C9—H9A	108.8	C19—C18—H18	119.3
C10—C9—H9A	108.8	C20—C19—C18	117.81 (13)
N1—C9—H9AB	108.8	C20—C19—C22	120.67 (13)
C10—C9—H9AB	108.8	C18—C19—C22	121.52 (13)
H9A—C9—H9AB	107.7	C21—C20—C19	121.14 (13)
C11—C10—C9	111.74 (11)	C21—C20—H20	119.4
C11—C10—H10A	109.3	C19—C20—H20	119.4
C9—C10—H10A	109.3	C20—C21—C16	121.33 (13)
C11—C10—H10B	109.3	C20—C21—H21	119.3
C9—C10—H10B	109.3	C16—C21—H21	119.3
H10A—C10—H10B	107.9	C19—C22—H22A	109.5
C12—C11—C10	113.46 (12)	C19—C22—H22B	109.5
C12—C11—H11A	108.9	H22A—C22—H22B	109.5
C10—C11—H11A	108.9	C19—C22—H22C	109.5
C12—C11—H11B	108.9	H22A—C22—H22C	109.5
C10—C11—H11B	108.9	H22B—C22—H22C	109.5
H11A—C11—H11B	107.7		
			
C8—S1—C1—C2	154.11 (11)	N1—C7—C8—S1	−35.27 (15)
C8—S1—C1—C6	−25.32 (12)	C1—S1—C8—C15	−139.90 (12)
C6—C1—C2—C3	1.0 (2)	C1—S1—C8—C7	44.53 (11)
S1—C1—C2—C3	−178.44 (11)	C7—N1—C9—C10	79.89 (15)
C1—C2—C3—C4	1.7 (2)	C6—N1—C9—C10	−96.17 (14)
C2—C3—C4—C5	−2.0 (2)	N1—C9—C10—C11	178.84 (12)
C3—C4—C5—C6	−0.3 (2)	C9—C10—C11—C12	172.66 (13)
C4—C5—C6—C1	2.9 (2)	C10—C11—C12—C13	−178.97 (14)
C4—C5—C6—N1	−174.94 (13)	C11—C12—C13—C14	−64.8 (2)
C2—C1—C6—C5	−3.2 (2)	C7—C8—C15—C16	−178.20 (12)
S1—C1—C6—C5	176.17 (10)	S1—C8—C15—C16	6.3 (2)
C2—C1—C6—N1	174.59 (12)	C8—C15—C16—C21	144.09 (15)
S1—C1—C6—N1	−5.99 (18)	C8—C15—C16—C17	−35.5 (2)
C7—N1—C6—C5	−156.27 (13)	C21—C16—C17—C18	−2.2 (2)
C9—N1—C6—C5	19.42 (18)	C15—C16—C17—C18	177.39 (13)
C7—N1—C6—C1	25.95 (19)	C16—C17—C18—C19	−0.6 (2)
C9—N1—C6—C1	−158.36 (12)	C17—C18—C19—C20	2.4 (2)
C6—N1—C7—O1	175.34 (12)	C17—C18—C19—C22	−177.10 (14)
C9—N1—C7—O1	−0.48 (18)	C18—C19—C20—C21	−1.3 (2)
C6—N1—C7—C8	−3.51 (19)	C22—C19—C20—C21	178.20 (14)
C9—N1—C7—C8	−179.33 (11)	C19—C20—C21—C16	−1.6 (2)
O1—C7—C8—C15	−30.00 (19)	C17—C16—C21—C20	3.3 (2)
N1—C7—C8—C15	148.85 (13)	C15—C16—C21—C20	−176.31 (13)
O1—C7—C8—S1	145.87 (11)		

### Hydrogen-bond geometry (Å, º)

**Table:**

*D*—H···*A*	*D*—H	H···*A*	*D*···*A*	*D*—H···*A*
C4—H4···O1^i^	0.95	2.42	3.349 (2)	168
C15—H15···O1^ii^	0.95	2.45	3.2977 (17)	148

Symmetry codes: (i) *x*+1, *y*, *z*; (ii) −*x*, −*y*+1, −*z*+1.
